# Discogenic Back Pain: Literature Review of Definition, Diagnosis, and Treatment

**DOI:** 10.1002/jbm4.10180

**Published:** 2019-03-04

**Authors:** Kengo Fujii, Masashi Yamazaki, James D Kang, Makarand V Risbud, Samuel K Cho, Sheeraz A Qureshi, Andrew C Hecht, James C Iatridis

**Affiliations:** ^1^ Leni & Peter W. May Department of Orthopaedics Icahn School of Medicine at Mount Sinai New York NY USA; ^2^ Department of Orthopaedic Surgery University of Tsukuba Tsukuba Japan; ^3^ Department of Orthopaedic Surgery Brigham and Women's Hospital Harvard Medical School Boston MA USA; ^4^ Department of Orthopaedic Surgery Sidney Kimmel Medical College Thomas Jefferson University Philadelphia PA USA; ^5^ Department of Orthopaedic Surgery Hospital for Special Surgery New York NY USA

**Keywords:** DISCOGENIC BACK PAIN, LOW BACK PAIN, INTERVERTEBRAL DISC, DIAGNOSTIC CRITERIA, DISC DEGENERATION

## Abstract

Discogenic back pain is multifactorial; hence, physicians often struggle to identify the underlying source of the pain. As a result, discogenic back pain is often hard to treat—even more so when clinical treatment strategies are of questionable efficacy. Based on a broad literature review, our aim was to define discogenic back pain into a series of more specific and interacting pathologies, and to highlight the need to develop novel approaches and treatment strategies for this challenging and unmet clinical need. Discogenic pain involves degenerative changes of the intervertebral disc, including structural defects that result in biomechanical instability and inflammation. These degenerative changes in intervertebral discs closely intersect with the peripheral and central nervous systems to cause nerve sensitization and ingrowth; eventually central sensitization results in a chronic pain condition. Existing imaging modalities are nonspecific to pain symptoms, whereas discography methods that are more specific have known comorbidities based on intervertebral disc puncture and injection. As a result, alternative noninvasive and specific diagnostic methods are needed to better diagnose and identify specific conditions and sources of pain that can be more directly treated. Currently, there are many treatments/interventions for discogenic back pain. Nevertheless, many surgical approaches for discogenic pain have limited efficacy, thus accentuating the need for the development of novel treatments. Regenerative therapies, such as biologics, cell‐based therapy, intervertebral disc repair, and gene‐based therapy, offer the most promise and have many advantages over current therapies. © 2019 The Authors. *JBMR Plus* Published by Wiley Periodicals, Inc. on behalf of American Society for Bone and Mineral Research

## Introduction

Low back pain (LBP) is one of the major clinical and socioeconomic global health burdens. The prevalence of LBP is reported to be 31%,^1^, ^2^ and lifetime prevalence is reported to be 60% to 80%.^1^ LBP is a multifactorial condition that includes physiological and psychological factors, as well as brain changes.^3^ Intervertebral disc (IVD) degeneration is a significant cause of pain in LBP patients.^4^, ^5^, ^6^, ^7^, ^8^ Discogenic back pain and axial back pain are terms commonly used to describe back pain associated with IVD degeneration without herniation, anatomical deformity, or other alternate clear causes of pain and disability. Spinal surgery is very effective in addressing spinal deformity, radicular pain from herniation, spinal stenosis, and spondylolisthesis among other conditions. In contrast, axial back pain is multifactorial without a clear source of pain, which can arise from the IVDs and associated structures of the motion segment, such as facet joints, ligaments, and spinal muscles.^9^, ^10^, ^11^, ^12^, ^13^, ^14^ Axial LBP that is thought to originate from disc degeneration (discogenic pain) therefore remains hard to define, diagnose, and treat. It commonly requires prolonged treatment, has mixed‐to‐poor surgical outcomes, and opioids are often prescribed.^15^ Many studies have demonstrated high sensitivity of pain to IVD pathologies on MRI including high‐intensity zones and Modic changes,^16^, ^17^ although this sensitivity is often not specific to pain presentation. The absence of IVD degeneration on MRI is associated with significantly reduced pain, making it a more specific measure.^16^ The presentation of pain also varies widely among patients, making disability a more important indication for spinal surgery. Currently, there is no widely accepted standard for discogenic pain.^18^ This lack of a uniform definition lies in part because IVD degeneration is hard to isolate and is commonly implicated in pathologies in adjacent spinal structures, making improved nomenclature and consensus on spine pathology definitions and diagnosis an important ongoing area for research.^19^, ^20^ Our aims here are (1) to review the available definitions of discogenic back pain, (2) to describe the diagnostic criteria for discogenic back pain, (3) to examine current treatments for discogenic back pain, and (4) to identify sources of discogenic back pain to provide potential research targets for future treatments.

## Categorization of back pain

**Figure 1:**
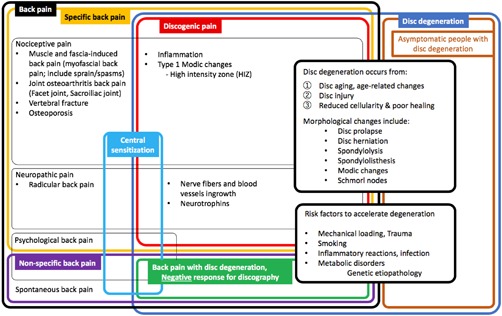
Categorization of back pain and disc degeneration conditions with elaboration on the origins of pain.

LBP has been categorized in many ways (Fig. 1). First, LBP can be divided into specific LBP and nonspecific LBP.^1^ Nonspecific LBP has been reported to account for 80% to 90% of overall LBP despite the recent progress in diagnostic tools such as radiography. In addition, treatment choices for chronic nonspecific LBP lack clarity; outcomes are often mixed because of the difficulty identifying the pain generator and multifactorial characteristics.^21^, ^22^ Specific pain includes nociceptive and neuropathic pain associated with muscle and fascia injury, spinal osteoarthritis, osteoporosis, and radicular back pain.^21^

Back pain can also be categorized by the origin of the pain: discogenic LBP, radicular back pain, facet joint osteoarthritis back pain, muscle and fascia‐induced back pain, and spontaneous occurring LBP.^20^ Discogenic pain can be categorized as a distinct category of back pain, mainly consisting of nociceptive and neuropathic pain (Fig. 1), although the specific causes of discogenic back pain are commonly multifactorial and can be challenging to diagnose and treat.

Muscle‐ and fascia‐induced back pain (myofascial back pain) is a type of pain that refers to a myofascial structure such as muscle and fascia. Its associated conditions include sprains, spasms, and contusions.^11^, ^23^, ^24^

Joint osteoarthritis back pain includes the facet joint and also the sacroiliac joint as one of the origins of LBP.^11^

Radicular back pain is a type of pain that radiates along the course of a spinal nerve root into the lower extremity. Radicular pain is caused by both nerve root compression and inflammation. Nerve root compression can occur in conditions including herniated disc, foraminal stenosis, peridural fibrosis, spondylolisthesis, and spondylolysis. Inflammatory cytokines are induced by herniated IVD and are considered to affect dorsal root ganglia to cause radiculopathy.^25^

Regardless of the cause of pain, LBP refers to a pain process of the central nervous system (CNS). Chronic LBP can result in permanent dysfunction of the CNS, with central sensitization considered to play an important role in chronic pain including hyperalgesia. Chronic pain conditions can be very difficult to address clinically because treatment of the spinal condition may not resolve the central sensitization.

## Definition of discogenic pain

Adams and Roughley proposed definitions for disc degeneration and degenerative disc disease as follows^26^:

“The process of disc degeneration is an aberrant, cell‐mediated response to progressive structural failure. A degenerate disc is one with structural failure combined with accelerated or advanced signs of aging. Early degenerative changes should refer to accelerated age‐related changes in a structurally intact disc. Degenerative disc disease should be applied to a degenerate disc that is also painful.” As with many other complex disease states, degenerative disc disease is helpful as an organizing principle for multiple complex pathologies, but is also confusing in its lack of specificity. Further clarification and definition of the terms “early degenerative changes” and “age‐related changes” will help in our understanding of the broader term “degenerative disc disease.”

Early degenerative changes usually occur and progress without symptoms; there are usually subtle changes to the matrix of the nucleus pulposus (NP) and inner annulus fibrosus (AF).^14^ As a result of these nonpainful conditions, it is hard or impossible to separate such early degenerative changes from aging in the human. However, basic science studies can discern early degenerative changes to involve a shift in the balance of anabolic and catabolic activities that can predispose to accelerated degeneration, as well as increased proinflammatory cytokine production from IVD cells that are considered to be nociceptive and noxious triggers that can progress to painful conditions.^27^, ^28^ For example, nitric oxide, leukotrienes, prostaglandin E, and lactic acid are known to increase in early IVD degeneration; all are considered to be powerful direct nociceptive stimuli.^14^ Disc degeneration is perhaps most easily distinguished by a loss of tissue and by structural derangement. Although disc degeneration is age‐associated, disc degeneration is not equal to disc aging, but rather involves pathological structural defects, which are distinct from age‐associated changes.^29^, ^30^ Such localized defects can result in strain concentrations, apoptosis, and increase proinflammatory conditions and deformities, which can result in painful conditions.^30^

Disc aging and age‐related changes occur in all spinal discs of all individuals, and though it correlates with IVD degeneration, it can be separated from the IVD degeneration processes (Table 1).^29^, ^31^, ^32^, ^33^ IVD aging is most commonly described as a loss of proteoglycans and water content in the NP. Vo and colleagues described three distinct phases of the biochemical cascade process of disc aging.^31^ The first phase is biomolecular damage, which includes free‐radical production, the accumulation of advanced glycation endproducts, epigenetic damage that results in oxidative stress, the loss of homeostasis, and extracellular matrix degradation with a loss of proteoglycans and hypo‐osmolality. The second phase is aberrant responses to damage. This phase includes cellular senescence and apoptosis, as well as dysregulated signaling (NF‐κB, mitogen‐activated protein kinases, and hypoxia‐inducible factor).^34^, ^35^, ^36^, ^37^ Functional and phenotypic changes in AF and NP cells occur as a consequence of accumulated biomolecular damage, and additionally lead to proteoglycan loss and dehydration of NP. The third phase is a loss of biologic structure and function, which includes a loss of disc matrix integrity, the loss of disc functional cells or stem cells, and a loss of disc biomechanics. Therefore, although aging is distinct from degeneration, it involves many conditions that can predispose to injury, inflammation, and frustrated healing conditions that are essential characteristics of degeneration.

**Table 1:** Factors to Distinguish Intervertebral Disc (IVD) Aging and IVD Degeneration in Their Early Stages

IVD aging	IVD degeneration
Loss of proteoglycans and water content Increased collagen crosslinking and advanced glycation end‐product accumulation	Proinflammatory cytokines, nociceptive stimuli (nitric oxide, leukotrienes, prostaglandin E, lactic acid)
Endplate sclerosis Hypo‐osmolarity and reduced nutrition Reduced cellularity and increased cellular senescense	Injury and neurovascular ingrowth Pathological structural defects Biomechanical dysfunction
Dysregulated nutrient sensing Signaling of NF‐κB, mitogen‐activated protein kinases	

Late IVD degeneration has features that can create painful responses; it is identifiable in clinical radiographic evaluation in humans and in animal models of degeneration. Late IVD degeneration includes disc height loss (disc space narrowing), osteophyte formation, internuclear calcification, endplate sclerosis as seen in plain radiographs and via signal decrease in T2‐weighted (T2W) MRI, a loss of AF/NP boundary, an irregular cartilage layer, and a selective loss of horizontal trabeculae in MRI.^32^, ^37^, ^38^, ^39^

**Figure 2:**
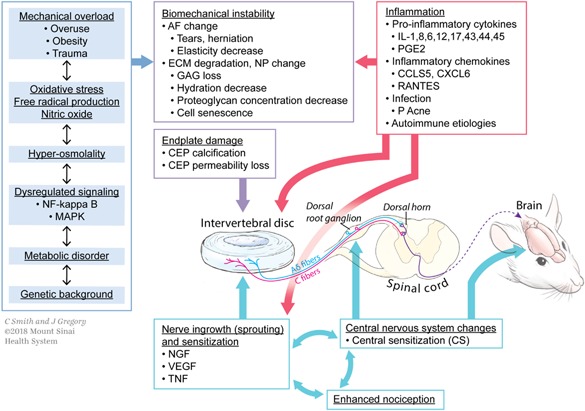
Origins of discogenic pain involving early and late degenerative changes of the intervertebral disc that interact with the peripheral and central nervous systems. The source of disc degeneration involves genetic predisposition, metabolic disorders, dysregulated signaling as well as mechanical overload, and oxidative stresses to drive the biomechanical injury, instability, and inflammation of degenerated discs. The interaction of intervertebral disc degeneration with the peripheral nervous system, including dorsal root ganglion, and with the central nervous system, including the dorsal horn of the spinal cord, can result in increased sensitization, nerve in growth, and central sensitization as discogenic pain advances from an acute to a chronic condition.

Discogenic pain therefore involves multifactorial changes occurring with late IVD degeneration that interact with the peripheral nervous system and the CNS to induce pain (Fig. 2). Pain can be the result of biomechanical instability, endplate damage, nerve ingrowth and sensitization, and inflammation. The causes of discogenic pain and late IVD degenerative changes are also multifactorial and can vary from mechanical overloading, oxidative stress, metabolic disorders, and genetics.^40^, ^41^, ^42^, ^43^, ^44^

## Currently known source of pain/cause of pain from degenerated IVD

Not all degenerated IVDs exhibit discogenic pain; however, IVD degeneration is no doubt among the most important key factors. IVD aging and age‐related changes, as well as IVD injury result in morphological changes including disc prolapse, disc herniation, spondylosis, spondylolisthesis, Modic changes, and Schmorl nodes (Fig. 1). Mechanical overload, oxidative stress, hyperosmolarity, dysregulated signaling, systemic metabolic disorder, and genetic polymorphisms contribute to the progression of structural change and biomechanical instability. Also, inflammation plays an important role in degenerative cascade. Nerve ingrowth, sensitization, and CNS changes are considered direct causes of discogenic pain. Numerous preclinical in vivo and in vitro studies are focused on these sources and causes of discogenic pain. To stop an irreversible cascade at some point and to regenerate to a healthy condition would be an ideal future treatment strategy.

## Diagnostic criteria for discogenic pain

Malik and colleagues performed a systematic review of the existing diagnostic criteria and treatments of discogenic pain, and developed a table listing the various consensus statements for the diagnosis of presumed discogenic pain.^12^ Consensus statements on the discogenic criteria for the diagnosis of discogenic pain have been made by the International Spine Intervention Society, the International Association for the Study of Pain, the North American Spine Society, and the American Society of Interventional Pain Physicians.^13^, ^45^, ^46^, ^47^ The modality to diagnose discogenic pain in all these criteria includes provocative discography or CT discography. The advantage of provocative discography is its relatively high specificity and sensitivity. However, clinical evidence indicates a high risk of accelerated disc degeneration and disc herniation in patients after discography; therefore, discography may be too invasive to use as a diagnostic procedure.^48^ Provocative discography also has a high false‐positive rate. As a result, the clinical use of discography should be limited to select patients who are planning a surgical procedure in the near future as a confirmatory step rather than as an early diagnostic procedure. Therefore, diagnostic methods other than discography are needed; currently there are no standard diagnostic methods.^16^, ^18^

## Noninvasive diagnostic tools for discogenic pain

Noninvasive diagnostic tools for discogenic pain include a clinical examination, pain diagrams/questionnaires, serum biomarkers, and MRI.

A clinical examination and history are important to properly diagnose back pain. Red flags that indicate the possibility of cancer, infection, or trauma must be identified or ruled out.^49^ Nonorganic signs or “Waddell signs” should be kept in mind to detect psychological distress.^49^ The localization of back pain is an important factor and can be derived from patient inquiry and simple clinical examination. Specifically, centralized pain has high sensitivity, but a poor specificity with regard to discogenic pain in the presence of a competent annulus, whereas lateralized pain patients often present without central pain and commonly have facet joint‐originated pathology.^49^, ^50^, ^51^

Serum biomarkers have been studied as a novel diagnostic tool for back pain. This is an emerging field and new biomarkers are being developed that can distinguish different sources of back pain. Serum biomarkers are quantitative and objective measurements, and can be used as indicators of biological processes involved in discogenic pain or as indicators for systematic disorders such as osteoporosis.^52^ Currently reported candidates for serum biomarkers for back pain include C‐C motif ligand 5, C‐X‐C motif ligand 6, IL‐6, high‐sensitivity C‐reactive protein, TNF‐α, IL‐1β, and type 2 collagen.^52^, ^53^, ^54^, ^55^, ^56^, ^57^, ^58^, ^59^ Matrix‐assisted laser desorption ionization time‐of‐flight mass spectrometry (MALDI‐TOF‐MS) has also been shown to distinguish discogenic LBP from the other forms of chronic back pain, and complement C3 and fibrinogen are potential serum biomarkers for discogenic back pain.^60^ Nevertheless, serum cytokines and other biomarkers can be from a variety of sources of systemic conditions, so their specificity to discogenic back pain conditions must be validated with other methods. In terms of local biomarkers, substance P, neurofilament, and vasoactive‐intestinal peptide immunoreactive nerve fibers in the painful discs have been shown to be more extensive than in control discs.^61^

In general, MRI and Pfirmann scoring are widely used to diagnose disc degeneration in clinical and basic research.

High‐intensity zone (HIZ) in MRI is a hyperintense signal in the posterior AF clearly dissociated from the signal of the NP. This bright area surrounded by a low‐intensity (black) signal of the AF is appreciably brighter than the CSF signal at the same level on sagittal T2W MRI of L1 to S1.^17^, ^62^, ^63^ HIZ has been shown to correlate with annulus damage and increased pain; it is also consistent with findings on discography.^64^, ^65^

Type 1 Modic changes have been shown to have high specificity for positive discography.^66^, ^67^, ^68^, ^69^ Modic changes are MRI signal‐intensity changes in the vertebral bone marrow that reflect lesions not related to malignancy, pyogenesis, or seropositive rheumatic disorders. There are three types of Modic changes based on appearance in T1W and T2W MRI.^68^, ^70^ The pathology of Modic change has been revealed to be a fibrogenic and proinflammatory crosstalk between bone marrow and adjacent discs. Type 1 Modic change is an MRI finding with hypointense signal on T1W sequences and hyperintense signal on T2W sequences; these changes also highly associate with IVD degeneration and back pain.^71^ The roles of bacterial infection and autoimmune etiologies have been described recently.^70^, ^72^, ^73^, ^74^

Conventional T2W MRI is a qualitative or semiqualitative tool to assess morphology and water content, and is not sensitive to proteoglycan content.^75^

There are more novel MRI techniques to quantify the biochemical changes and to identify potential biomarkers for discogenic pain. Recent studies have shown a relationship between low pH with discogenic pain; hence, pH may be a metabolic biomarker for discogenic pain.^76^, ^77^ Chemical exchange saturation transfer (CEST) is a technique to measure pH‐dependent signal changes that are known to be important in degeneration; quantitative chemical exchange saturation transfer MRI (qCEST MRI) also has the potential to detect pH changes in IVDs.^78^ In addition, the ratio of R1ρ dispersion to CEST is also reported to have the potential to detect painful IVDs.^79^ T2W MRI values can be used for the quantification of moisture content by T2 mapping; T2 mapping can be used as a quantitative diagnostic tool in disc degeneration and discogenic pain. MRI T2 relaxation time is a quantitative parameter that is sensitive to changes in collagen and water content in IVD. Also, MRI T1ρ is associated with a loss of macromolecules and may be sensitive to early biochemical changes in IVD degeneration.^80^, ^81^ Delayed gadolinium‐enhanced MRI of cartilage (dGEMRIC) is a quantitative analysis of sulfated glycosaminoglycans with cartilaginous tissue that has been applied to IVD.^82^ High‐resolution magic angle spinning NMR spectroscopy (HR‐MAS spectroscopy) is a qualitative and quantitative analytic method previously used to detect collagen breakdown and the decreased concentration of NP proteoglycans.^83^ To assess cartilage integrity, the measurement of fixed‐charge density of cartilage by sodium MRI (23Na MRI) has been developed and may have applications in IVD.^84^ Ultrashort time‐to‐echo (UTE) MRI assesses the MRI signal from short‐T2 components that are not detected on conventional T2W MRI; hyper‐ or hypointensity changes located within the disc are reported to associate with LBP and disability in comparison with traditional T2W MRI.^85^ These methods can be used to diagnose the disease in its early stages, but still need more investigation and validation.^86^

In summary, the currently available noninvasive diagnostic tools for discogenic pain, including the clinical examination, will always play important roles in the diagnostic process; however, MRI findings including type 1 Modic changes and HIZ seem especially useful. Phenotypic definitions and novel imaging methods are also being developed for IVD degeneration to make MRI diagnosis precise and useful. However, at present the specificity/sensitivity on MRI findings is not sufficient and shows only a moderate effectiveness in fully identifying a source of pain.^74^, ^87^ Diagnostic methods with the specificity of discography are in need of development to detect painful lesions. MRI sequences, such as T1ρ, have the potential to be a strong clinical tool, but the evidence and proof of validity are not established yet. Serum biomarkers also have the potential to be a useful supportive diagnostic tool in LBP.

## Available clinical treatments for discogenic pain

Reviews of evidence‐based guidelines describe consistent recommendations and guidance for the evaluation of chronic LBP, but there is a lack of clarity on treatment recommendations.^88^

### Invasive treatment: surgeries

Fusion surgeries remove the painful disc itself and prevent the recurrence of discogenic pain by eliminating the mobile parts of the spine to prevent instability from occurring again.^61^ The disadvantages of fusion surgery are (1) its invasiveness, (2) complications as a result of major surgery, and (3) adjacent segment disorder (ASD), which can accelerate degeneration because of excess loading on the discs adjacent to the fusion.^88^, ^89^, ^90^, ^91^, ^92^ Total disc replacement (TDR) has less of a risk of ASD compared with fusion surgeries; however, there are known risk factors including heterotopic ossification and the risk of reoperation. TDR is best indicated in spinal levels (eg, cervical) where maintained mobility is of greater importance.^93^, ^94^, ^95^, ^96^, ^97^ Percutaneous endoscopic or minimally invasive tubular decompressive surgeries are also widely performed; good clinical results have been reported in selected patients with a localized lesion and IVD herniation.^98^, ^99^, ^100^ However, obtaining successful clinical outcomes for treating discogenic LBP with these surgical measures have been challenging.

### Semi‐invasive treatment: injection, intradiscal procedures

Thermal intradiscal/annular techniques (intradiscal electrothermal therapy) use radiofrequency probes to treat painful lesions, usually with intradiscal insertion into the posterior wall of the IVD. These methods destroy inflammatory or painful tissue/mediators and newly formed nerve fiber in the IVD; the heating also seals the AF tears by shrinking them.^101^, ^102^, ^103^, ^104^, ^105^ These techniques have mostly been abandoned because of relatively poor outcomes.^106^, ^107^

Electrostimulation is a therapy used for intractable chronic neuropathic pain.^104^ The spinal cord stimulation system consists of an electrical lead placed in the epidural space and an implanted pulse‐generating battery system. The electrical pulse transmitted from the lead is intended to produce a nonpainful paresthesia overlapping the patient's areas of pain, and overlapping paresthesias to the painful areas correlate with optimal pain relief. However, there exists a relatively high adverse‐event occurrence rate of up to 34.3%, including infection, surgical site pain, dural puncture, and equipment/system problems.^104^ Our limited experience also suggests that it is not long‐lasting.

Epidural injection therapy, including steroid and anesthetics, has been widely used clinically. Epidural injection can be delivered through three different anatomical routes: caudal, interlaminar, and transforaminal.^98^, ^108^, ^109^ This therapy is used mainly for lumbar radiculopathy, but is also used for LBP.^104^, ^110^, ^111^ However, its long‐term efficacy is still unknown.^104^, ^105^, ^112^, ^113^, ^114^

Methylene blue has been tried for intradiscal injection, to chemically ablate nerve endings.^115^, ^116^ Methylene blue injection into the epidural space has been tried in animal models, but not introduced for human use because of its potential neurotoxic effects.^117^, ^118^, ^119^ Current spinal or intradiscal injections and procedures have no demonstrated effects on IVD regeneration, and are not able to completely stop the progression of the degeneration cascade.

### Regenerative medicine

Regenerative therapies show promise with numerous basic studies concentrating on regenerative medicine of the IVD. The avascular nature of the IVD creates a hypoxic microenvironment with relatively low cellularity and slow cell metabolic rates, which present a challenge for reversing IVD degeneration and regenerating spinal tissues.^120^ As a result, regenerative medicine strategies are considered most promising for early IVD degeneration, where there is still the possibility to promote repair and healing of the IVD. However, it may be more realistic to target slowing the progressive degeneration process or otherwise deliver cells and/or drugs to reduce the potential for painful degeneration. At present, the clinical application of regenerative medicine strategies is limited to mesenchymal stem cell (MSC)/bone marrow aspirate, platelet‐rich plasma (PRP), and chondrocytes. The number of studies and the number of patients recruited are also limited.^121^, ^122^ With regards to MSC therapy, feasibility and safety have been suggested in pilot clinical studies among chronic back pain patients.^123^, ^124^, ^125^ Analgesic effect, functional improvement, and increased water content by MSC injection therapy have also been reported, although there was no effect on recovering disc height.^123^, ^124^, ^125^ Furthermore, improvement may be restricted to a group of responders.^124^, ^125^ Optimization of indication (to distinguish responders and nonresponders), cell‐source, cell concentration, and scaffold type are needed, as are larger‐scale clinical trials to show long‐term safety and efficacy. Intradiscal PRP injection seems safe; however, its efficacy remains unclear.^126^, ^127^, ^128^, ^129^ Chondrocyte transplantation studies showed some positive results in a small number of participants; further studies are needed to show efficacy.^130^, ^131^, ^132^ Experimental studies demonstrated cell leakage following injection with ectopic calcifications as a potential complication, thus motivating the need for a cell carrier to help enhance cell injections.^133^, ^134^

IVD repair techniques or implant biomaterials for annulus closures and NP replacement have the potential to repair the IVD, yet also create a risk for reherniation, which can cause nerve compression and the possibility of a recurrent painful condition or worsening of a condition. As a result, especially after discectomy, IVD repair/closure techniques have been developed to prevent reherniation and consequent progression of IVD degeneration. Some annular closure devices and NP replacement devices have been introduced clinically, including Barricaid (Intrinsic Therapeutics, Woburn, MA, USA), Xclose Tissue Repair System (Anulex Technologies, Minnetonka, MN), Inclose Surgical Mesh System (Anulex Technologies, Inc., Minnetonka, MN), NuCore® Injectable Nucleus hydrogel (Spine Wave, Inc., Shelton, CT, USA), NeuDIsc (Replication Medical, Inc., Cranberry, NJ), DiscCell (Gentis, Wayne, Pennsylvania), DASCOR Disc Arthroplasty System (Disc Dynamics, Inc., Eden Prairie, Minnesota), BioDisc (CryoLife, Atlanta, Georgia), and NucleoFix (Replication Medical, Inc., Cranberry, NJ).^135^ The Barricaid AF closure device showed midterm clinical feasibility, efficacy, and safety.^136^ Although an FDA panel confirmed its efficacy in preventing reherniations following discectomy, the presence of lytic endplate lesions and device subsidence raised safety concerns.^137^ AF repair and NP replacement biomaterials and devices remain an emergent area of research as described in multiple excellent recent studies and reviews.^132^, ^134^, ^138^, ^139^, ^140^, ^141^ These biomaterials must show efficacy for improved spinal healing or pain reduction, while also avoiding reherniation and/or adjacent tissue damage.

Whole IVD tissue‐engineering strategies have also been considered as an alternative to spinal fusion. Whole IVD repair strategies are more ambitious than AF repair or NP replacement, yet the possibility of designing an integrated whole IVD structure offers opportunities to reduce herniation risk and to promote integration with the vertebral endplate. Several total‐disc tissue‐engineered replacements are being developed and evaluated in small and large animal models.^138^, ^142^, ^143^, ^144^, ^145^, ^146^, ^147^

In summary, some clinical studies of regenerative medicine strategies have shown positive results; additional long‐term more‐powered high‐quality studies showing their efficacy and safety are desired. Several biomaterials are being developed for cell delivery, AF repair, and NP replacement to promote IVD repair, and whole‐tissue‐engineered structures are being developed to replace for spinal fusion or total disc arthroplasty.

### Nonoperative management

Nonoperative management of discogenic LBP includes oral analgesics, physiotherapy, psychotherapy, and acupuncture/dry needling.^105^, ^147^

Oral pharmaceutical management for analgesic purposes includes acetaminophen, NSAIDs, skeletal muscle relaxants, tramadol, steroids, and opioids. Opioids are frequently prescribed for back pain patients; however, because of their physical dependence and tolerance, long‐term opioid use is discouraged.^148^, ^149^, ^150^, ^151^

Animal studies have shown potential benefits of traction therapy; however, the randomized controlled human trial showed no significant differences in clinical outcomes.^152^, ^153^, ^154^, ^155^ The McKenzie Method of Mechanical Diagnosis and Therapy is a famous treatment for LBP; it consists of a classification system and classification‐based physical therapy.^156^, ^157^ However, there is still limited evidence for the efficacy of the McKenzie method in chronic LBP.^158^, ^159^

Cognitive‐behavior therapy (CBT) is a form of psychotherapy and is effective for various problems, including chronic LBP. Structured CBTs have been shown to be effective; nevertheless, psychological interventions should be performed with suitable physiotherapy.^147^, ^160^, ^161^, ^162^

Acupuncture/dry needling is an option for discogenic pain, and a systematic review showed its effectiveness for pain relief and functional improvement compared with no treatment or sham in the short term (6 to 12 weeks). However, it is not more effective than any other treatments.^163^

Nonoperative management strategies have demonstrated good results, and some systematic reviews have shown even comparative clinical results to surgical treatment.^105^, ^147^ On the other hand, opioid use should be restrictive because of the risk of developing opioid‐induced hyperalgesia, abuse, and misuse.^150^

### Summary and guideline recommendations of currently available clinical treatments for discogenic pain

Clinical treatment options for chronic LBP were recently reviewed by Foster and colleagues, with guidelines for the management of LBP mostly focusing on nonoperative management.^164^ Patients are advised against bed rest and to stay active or to engage in nonspecific back exercises for first‐line treatment.^88^, ^164^ Psychological therapies including cognitive‐behavior therapy are also consistently recommended in guidelines.^164^, ^165^, ^166^, ^167^ Other nonoperative management techniques such as manipulation, acupuncture, and interdisciplinary rehabilitation (physical therapy) are categorized as second‐line treatment for patients who have not responded to first‐line treatments.^168^ Oral pharmaceutical management is recommended only following an inadequate response to first‐line nonpharmaceutical treatments. Epidural injections are not recommended in recent guidelines, although epidural injections for severe radicular pain are recommended.^164^, ^165^, ^166^, ^167^ Fusion surgeries are categorized as second‐line or adjunctive treatment options, whereas TDRs do not have strong long‐term outcome data yet.

## Summary

In this review, we defined the clinical entity of discogenic pain as multifactorial changes occurring with late IVD degeneration that interact with the peripheral nervous system and the CNS to induce painful conditions. We also described diagnostic criteria and current treatments of discogenic pain based on a broad clinical literature review, and identified sources of discogenic pain from the literature to highlight potential research targets. Discogenic pain encompasses multiple conditions associated with IVD degeneration, which makes a single definition limiting. Improved diagnostic methods are helping to more precisely define specific pathologies associated with this broad condition. The most specific existing diagnostic criteria, such as discography, are invasive with evidence suggesting it can lead to accelerated IVD degeneration. Clinicians therefore need alternative noninvasive diagnostic methods that more precisely characterize IVD degenerative conditions and their specific relationships with the progression of painful conditions to better inform treatment options. There are a number of treatments/interventions for discogenic back pain currently. The lack of a specific diagnosis makes nonoperative management the most important first‐line treatment. Regenerative therapies, such as biologics, cell‐based therapy, IVD repair, and gene‐based therapy, are minimally invasive interventions that will likely have advantages over more‐invasive current surgical approaches once safety, reliability, and efficacy are shown in human studies.

## Disclosures

All authors state that they have no conflicts of interest.
